# Analysis of evolutionary patterns of genes in *Campylobacter jejuni* and *C. coli*

**DOI:** 10.1186/2042-5783-2-8

**Published:** 2012-08-28

**Authors:** Lars Snipen, Trudy M Wassenaar, Eric Altermann, Jonathan Olson, Sophia Kathariou, Karin Lagesen, Monica Takamiya, Susanne Knøchel, David W Ussery, Richard J Meinersmann

**Affiliations:** 1 Department of Chemistry, Biotechnology and Food Sciences, Norwegian University of Life Sciences, Ås, Norway; 2, Molecular Microbiology and Genomics Consultants, Zotzenheim, Germany; 3 Ruminant Nutrition and Microbiology, AgResearch Limited, Grasslands Research Centre, Palmerston North, New Zealand; 4 Department of Microbiology, North Carolina State University, Raleigh, North Carolina, USA; 5 Department of Food, Bioprocessing & Nutrition Sciences, North Caroline State University, North Carolina, Raleigh, USA; 6 Centre for Ecological and Evolutionary Synthesis (CEES), University of Oslo, Dept. of Biology, P.O. Box 1066 Blindern 0316 Oslo, Norway and Centre for Molecular Biology and Neuroscience, Institute of Medical Microbiology, Oslo University Hospital, Oslo, Norway; 7 Department of Food Science, Faculty of Life Sciences, Copenhagen University, Copenhagen, Denmark; 8 Centre for Biological Sequence Analysis, Technical University of Denmark, Lyngby, Denmark; 9 Bacterial Epidemiology and Antimicrobial Resistance Research Unit, Richard B. Russell Agricultural Research Center, Agricultural Research Service, U.S. Department of Agriculture, Athens, Georgia, USA; 10, Riddet Institute, hosted by Massey University, Palmerston North, Private Bag 11222, New Zealand

## Abstract

**Background:**

The thermophilic *Campylobacter jejuni* and *Campylobacter coli* are considered weakly clonal populations where incongruences between genetic markers are assumed to be due to random horizontal transfer of genomic DNA. In order to investigate the population genetics structure we extracted a set of 1180 core gene families (CGF) from 27 sequenced genomes of *C. jejuni* and *C. coli*. We adopted a principal component analysis (PCA) on the normalized evolutionary distances in order to reveal any patterns in the evolutionary signals contained within the various CGFs.

**Results:**

The analysis indicates that the conserved genes in *Campylobacter* show at least two, possibly five, distinct patterns of evolutionary signals, seen as clusters in the score-space of our PCA. The dominant underlying factor separating the core genes is the ability to distinguish *C. jejuni* from *C. coli*. The genes in the clusters outside the main gene group have a strong tendency of being chromosomal neighbors, which is natural if they share a common evolutionary history. Also, the most distinct cluster outside the main group is enriched with genes under positive selection and displays larger than average recombination rates.

**Conclusions:**

The *Campylobacter* genomes investigated here show that subsets of conserved genes differ from each other in a more systematic way than expected by random horizontal transfer, and is consistent with differences in selection pressure acting on different genes. These findings are indications of a population of bacteria characterized by genomes with a mixture of evolutionary patterns.

## Background

Bacterial populations are judged to be clonal based on the degree of linkage disequilibrium that is observed in the evolution of various loci on the genome. Population genetics, which studies the flow of genes within and between populations, has been applied to bacteria with the goal of finding the genes that are either shared between various subpopulations, or which distinguish between them. Population genetics is best performed by the analysis of discrete characters, for which DNA sequence data are optimal. Sequencing of entire bacterial genomes is on the horizon for being practical on a routine basis, but meaningful analyses of the data is lagging. For this reason, multilocus sequence typing (MLST), which was developed by determining the partial DNA sequence of each of a selected number of housekeeping genes [1], will continue to guide the approach to analysis. Multiple gene fragments at different positions of the chromosome are selected to represent an entire genome. Ideally, the genes that are selected should not be under selective pressures that affect their migration. It turns out that in many species of bacteria, including *Campylobacter jejuni* as a prime example, the housekeeping genes are subject to horizontal gene transfers (HGT), which are recognized as recombination events [2]. Thus the flow of individual genes may not be representative of the migration or evolution of the bacterial lineage, if a lineage can be defined at all.

*C. jejuni* is a leading bacterial cause of human diarrheal disease in most developed countries [3]. This has motivated research on tracking the sources of this zoonotic agent and its close cousin, *Campylobacter coli*. Consumption or handling of poultry products is recognized as the predominant risk factor for infection with *C. jejuni*, with exposure to pets and water, or the use of proton inhibitors, as additional significant contributors [4]. The full transmission cycle of these two pathogens is still unresolved and its identification is complicated by the wide genetic diversity observed within these species. Phenotypic and genotypic characterization of *C. jejuni* and *C. coli* isolates from various sources has not resulted in an unequivocal understanding of their transmission routes.

When HGT is absent or rare, a lineage can be defined by all, or the majority, of conserved genes in a genome. When a limited set of genes are affected by HGT, the population structure will show a mixture of evolutionary patterns, which was defined as a meroclone by Milkman [5], and the conserved portion of the genome was referred to as the clonal frame [6]. On the other hand, when HGT occurs at rates that are low enough so that recent clonal associations can be observed, a weakly clonal population structure can be recognized. A weakly clonal population does not imply any grouping of genes involved in HGT, but is characterized by the frequency of HGT, which must occur frequently enough to be detected but not so frequently that the genome is panmictic [7]. If the history could be accurately put together for a long enough period, every gene in a weakly clonal population should have some evidence of recombination but with a random distribution of how recently it occurred, with the possibility of multiple hits in some genes [8]. By MLST, *C. jejuni* and *C. coli* have been interpreted to have a weakly-clonal population structures, with evidence for limited HGT between the two species [9-11].

The distinction between a meroclonal and a weakly clonal population structure can be determined more precisely by total genome analysis of a population. There are now enough genome sequences of *C. jejuni* and *C. coli* available to analyze all genes that are shared by all sequenced isolates, instead of the selection of seven genes typically used in MLST. Using 23 publicly available genome sequences and four additional unpublished genomes, our objective was to determine whether *C. jejuni* and *C. coli* adhere to the meroclonal or the weakly clonal model of lineage development. The basis behind this analysis is the assumption that fragments of DNA that have evolved together will have congruent phylogenies. In a weakly clonal population there should be one major phylogeny, and all incongruences should be random deviations from any pattern correlating with selection. In a meroclonal population we expect to see a mixture of several phylogenies, i.e. clusters of genes sharing some common evolutionary pattern. We have searched for congruent phylogenies by principal component analysis (PCA) on all normalized pairwise evolutionary distances. It was hypothesized that if the PCA analysis did segregate loci with congruent phylogenies, other observable factors affecting evolution should correlate with the observed clustering.

## Results

### Identifying core gene families

**Figure 1:**
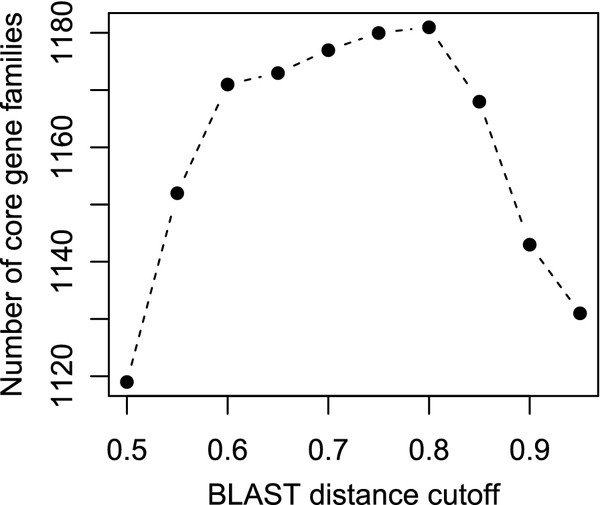
**Detection of core gene families.** The number of computed core gene families as a function of the BLAST distance cutoff.

The complete genome sequences of 22 *Campylobacter jejuni* and five *C. coli* were analyzed, see Table 1 for an overview. A set of core gene families (CGFs) was defined, based on BLASTP comparisons and hierarchical clustering using the distance metric as described in the Methods. Each defined CGF contained one gene member from each of the 27 genomes. In Figure 1 is shown how the choice of BLAST distance cutoff (see Methods section) affects the number of CGFs found. We decided to use the cutoff 0.8, giving the largest number of CGFs (1180), increasing the probability of observing interesting evolutionary patterns.

**Table 1:** Genomes used in this study

Genome	Size	Contigs	Genes	MLST	Source
jejuni subsp. jejuni NCTC11168	1.64	1	1658	ST43 (CC-21)	[12]
jejuni subsp. jejuni RM1221	1.78	1	1877	ST354 (CC-354)	[13]
jejuni subsp. jejuni 81116	1.63	1	1617	ST267 (CC-283)	[14]
jejuni subsp. jejuni 81-176 (TIGR)	1.70	1	1726	ST604 (CC-42)	TIGR
jejuni subsp. jejuni 81-176 (Yale)	1.62	1	1730	ST604 (CC-42)	Yale University
jejuni subsp. jejuni 84-25	1.67	5	1727	ST21 (CC-21)	TIGR
jejuni subsp. jejuni 260.94	1.66	10	1696	ST362 (CC-362)	TIGR
jejuni subsp. jejuni CG8486	1.60	19	1822	ST2943 (CC-574)	NMRC
jejuni subsp. jejuni CG8421	1.61	20	1747	ST1919 (CC-52)	NMRC
jejuni subsp. jejuni HB93-13	1.69	35	1727	ST22 (CC-22)	TIGR
jejuni subsp. jejuni 11601MD	1.74	1	1846	New ST (-)	NCSU
jejuni subsp. jejuni 1336	1.70	1	1755	ST841 (-)	University of Liverpool
jejuni subsp. jejuni 414	1.71	1	1806	ST3704 (-)	University of Liverpool
jejuni subsp. jejuni M1	1.62	1	1638	ST137 (CC-45)	DTU
jejuni subsp. jejuni IA3902	1.64	1	1651	ST21 (CC-21)	Iowa State University
jejuni subsp. jejuni CF93-6	1.68	14	1742	ST883 (CC-21)	TIGR
jejuni subsp. jejuni 327	1.62	48	1776	ST230 (CC-45)	UCPH
jejuni subsp. jejuni DFVF1099	1.73	71	1954	ST21 (CC-21)	UCPH
jejuni subsp. jejuni 305	1.81	333	2260	- (-)	UCPH
jejuni subsp. jejuni ICDCCJ07001	1.69	1	1802	ST986 (-)	[15]
jejuni subsp. jejuni S3	1.71	1	1765	ST354 (CC-354)	[16]
jejuni subsp. doylei 269.97	1.85	1	1982	ST1845 (-)	TIGR
coli RM2228	1.68	1	1715	ST1063 (CC-828)	TIGR
coli 6461	1.79	1	1885	- (CC-828)	NCSU
coli 11601	1.96	1	2091	ST1149 (CC-282)	NCSU
coli 6067	1.70	1	1786	ST1150 (CC-1150)	NCSU
coli JV20	1.71	34	1742	ST860 (CC-828)	Baylor college

Size of each *Campylobacter* genome (Mb) is followed by the number of contigs for each genome project, the number of predicted genes in our study, the MLST sequence type, the clonal complex and the source of the genome. Abbreviations: TIGR: The Institute for Genomic Research / J.C. Venter Institute; NMRC: Naval Medical Research Center; NCSU: North Carolina State University UCPH: University of Copenhagen; DTU: Danish Technical University.

We assessed whether the seven housekeeping genes most frequently used for MLST of *Campylobacter jejuni* and *C. coli* (*uncA*, *glnA*, *gltA*, *pgm*, *tkt*, *glyA* and *aspA*) were part of the CGFs, which indeed they were. Another much used marker, PorA, was also found in one CGF, while a second marker, *fla*, was not. The complete *fla* was not found in all draft genomes, but will most likely be detected once the genomes are completed.

### Principal components

**Figure 2:**
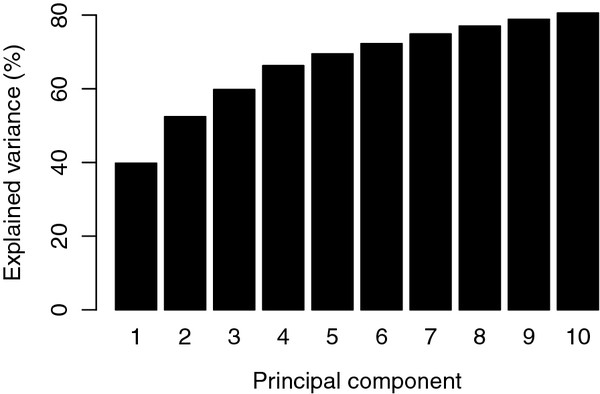
**Principal component analysis.** The cumulative explained variance in the principal component analysis of the evolutionary distance matrix. After three components more than 60% of the total variance is captured. Only the first 10 out of 351 components are shown.

The normalized evolutionary distance matrix *X* was used as a multivariate data set, as explained in the Methods section. A principal component analysis was performed on this data matrix. Figure 2 shows the cumulative sum of explained variance over the first 10 components. The first direction accounts for 40% of the variance in normalized evolutionary distances, and including the three first components we capture 60% of the variance. The remaining components contribute with gradually decreasing variance, and we assume this smaller variation is mostly unimportant and proceed with the downstream analysis in the three-dimensional space spanned by the first three components.

**Figure 3:**
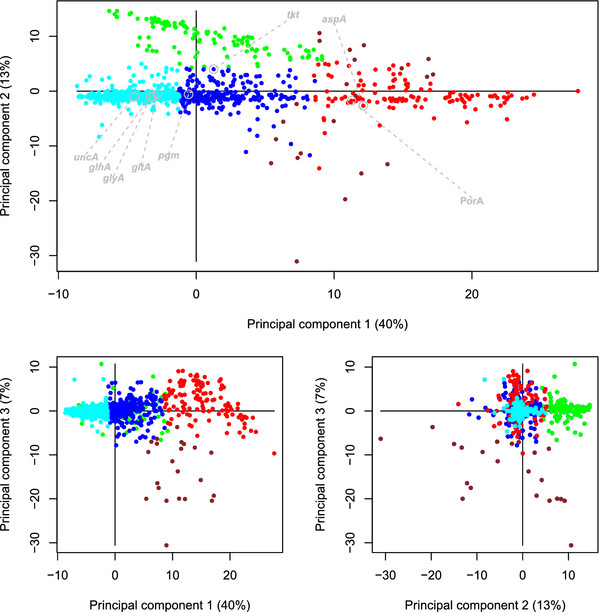
**Evolutionary space.** Every core gene of *Campylobacter* is represented as a dot in the space spanned by the three first principal components in our analysis. Instead of a three-dimensional plot we have used three pairwise scatter-plots, the upper panel being the most important (component 1 and 2). Explained variance is a measure of importance of the components, and is given by percentages of the total on each axis. The seven MLST-genes as well as the marker PorA are indicated in the upper panel only. Genes that are close to each other in this space show similar values for the 351 normalized pairwise evolutionary distances. Partitioning the CGFs into *K*=5 clusters leaves us with the clustering shown by the coloring of the dots. The blue and the cyan groups are both making up the central group of the CGFs, containing 935 of the 1180 *Campylobacter* core gene families. The red cluster, containing 120 CGFs, is separated from the blue along PC1. The green group (103 CGFs) is separated along PC2 and the scattered brown group (22 CGFs) mainly along PC3.

**Figure 4:**
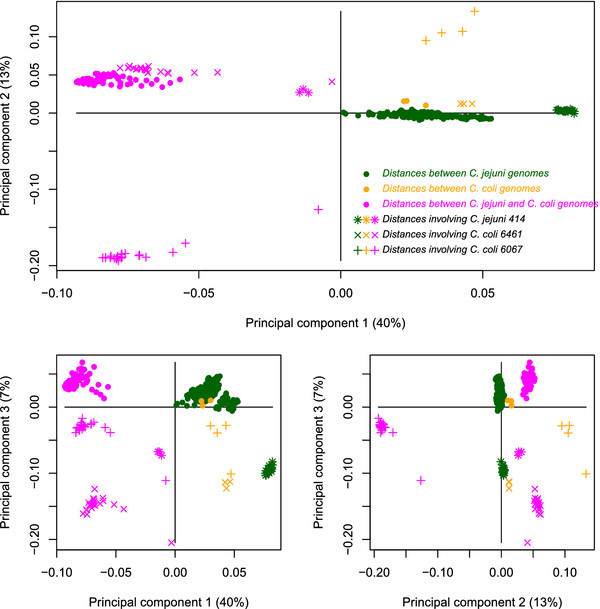
**PCA loadings.** The loadingplot for the three first principal components, corresponding to Figure 3. Each marker corresponds to an evolutionary distance between two genomes (351 markers). The upper panel legend indicates what the various markers and colors mean. Distances close to each other in this space show similar values across all core genes.

Figure 3 shows how each CGF corresponds to a point in the space spanned by the three first principal components, shown as three pairwise scatterplots. Each dot corresponds to a CGF, and those who are found close to each other will have similar normalized evolutionary distances, as explained in the Methods section. The upper panel is the most important, since this involves the two first components. Five of the seven MLST-genes are found in the dense region where most CGFs are found, while the markers *tkt* and especially *aspA* are found in different regions in the upper panel. The marker PorA is also very close to *aspA* in this space. The coloring is explained below. In Figure 4 we show the corresponding loadings for this PCA. The loadings indicate how the original 351 variables (pairwise distances) are related to the principal components, and this plot is included to help understand the components. From the upper panel of Figure 4 we see that the first principal component (horizontal axis) is spanned by within-species distances (darkgreen/orange markers on the right) versus between-species distances (magenta markers on the left). The big picture emerging from all core genes is the separation between *jejuni* and *coli*. The second component (vertical axis in upper panel or horizontal axis in lower right panel) seems to be spanned by all distances to the strain *coli* 6067 (’+’ markers). Likewise, the third component (vertical axes in lower panels) are mainly affected by the distances to the strains *coli* 6461 and *jejuni* 414 (’x’ and ’*’ markers).

### Clustering

**Figure 5:**
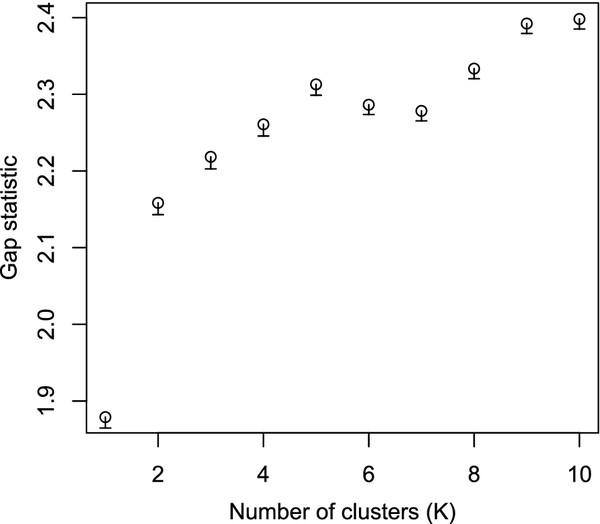
**Clustering gap-statistic.** The plot is a display of the gap-statistic values (100 simulations for each *K*-value) for each choice of number of clusters from *K*=1 (all CGFs belong to the same cluster) up to *K*=10. The error bars under each circle is the standard error for each value. The optimal number of clusters is where we see the first significant drop in gap-statistic value, i.e. where the gap statistic value *G*(*K*) is such that *G*(*K*)>*G*(*K* + 1)−*SE*(*K* + 1), where *SE*(*K* + 1) is the standard error at *K* + 1.

**Figure 6:**
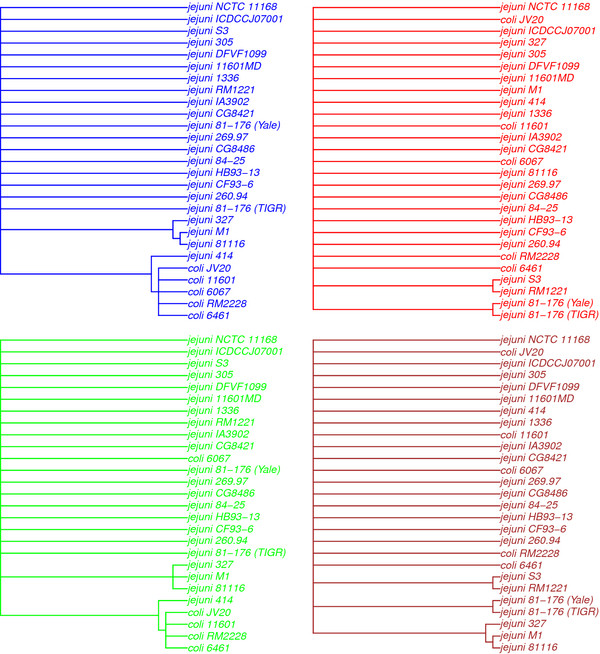
**Consensus trees.** For each of the clusters in Figure 3 we computed the consensus-tree based on the evolutionary distances, using the neighbor joining method. The groups colored in blue and cyan in Figure 3 have been merged into one big group here, and the blue tree in the upper left panel is the corresponding result. The other colors of the dots in Figure 3 corresponds to the colors of the trees here.

In Figure 5 we show the gap-statistic results for partitioning the CGFs into *K*=1,2,…,10 clusters. After *K*=5 we have the first significant drop in the gap-statistic, indicating that the data supports a split of the CGFs into 5 different clusters. The coloring of the dots in Figure 3 indicates the clusters. In Figure 6 we present the consensus-trees for each of the groups. Here we merged the blue and cyan cluster from Figure 3 into one big blue group. Figure 3 and 6 are alternative illustrations of the same gene groups. The big blue group has a tree where all 5 *C. coli* genomes are separated from the *C. jejuni* genomes, and *C. jejuni* 414 which is part of the same branch. In the red group the *C. coli* and *C. jejuni* genomes are not separated at all, in fact the branching is completely different from that of the blue tree. The green group is quite similar to the blue, but *C. coli* 6067 is no longer in the *C. coli*-branch of the green tree. The brown group, consisting only of 22 CGFs, is quite similar to the red tree, but with one branch similar to the blue tree.

### Gene features

Genes with a similar evolutionary history are often found to be located close to each other on the genome. Our analysis is not guided by this information, but in order to verify the clusters found by PCA, we made a brief investigation of positional distribution. In Table 2 we present the clumping index, as described in the Methods section, for each group. A value above 1.0 is an indication of clumping of the genes along the chromosome. Especially the red, green and brown clusters have indices much larger than 1.0.

**Table 2:** Results for the clusters identified

Cluster	Number	Clumping	CGF under	Recombination
	of CGF	index *I*	selection	rate $\bar{\gamma }$ (p)
Blue+cyan	935	1.73	2	27.3 (-)
Red	120	4.76	15	49.7 (<0.01)
Green	103	5.40	0	33.1 (0.02)
Brown	22	16.67	13	25.4 (0.70)

The clumping index *I*is described in Methods. CGFs under selection are the number of genes having a significantly negative Tajima *D*statistic. The recombination rate is the average for the cluster, and the associated p-value listed in parenthesis is for the t-test of difference to the blue+cyan cluster.

Table 2 also shows that the red and the brown cluster is highly enriched in genes under selection. In total 30 out of the 1180 CGFs had a significantly negative Tajima’s D statistic, and 28 of these 30 CGFs are found inside these two groups (15 in the red cluster, 13 in the brown).

**Figure 7:**
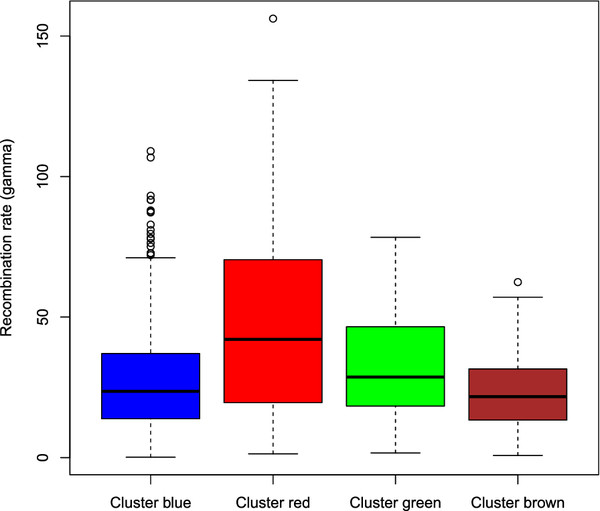
**Recombination rates.** For each CGF we computed the recombination rate *γ*and the box and whisker plot shows its distribution for each of the colored clusters in Figure 3, where the blue and cyan group has been merged into a big blue cluster. For each box the central line is the median, the box covers the interquartile range of the data, the whiskers cover the most extreme data points no more than 1.5 times the box width from the box edges and any data points more extreme than this are shown as individual circles.

The box and whisker plot of Figure 7 shows how the recombination rate *γ*for the different CGFs is distributed in each of the clusters. Especially the red cluster has a significantly elevated level of recombination rates. A simple analysis of variance using the *γ* values as response and the cluster membership as factor revealed that the red cluster has a significantly higher recombination rate than the blue cluster (*p*<0.01, see Table 2 for details).

## Discussion

This study is based on the identification of 1180 gene families present in 27 genomes of *Campylobacter jejuni* and *C. coli*, identified using a cutoff of 0.8 BLASTP distance, as defined in the Methods section. This cutoff is relatively permissive, allowing proteins that only share 20% amino acid similarity to appear in the same gene family. As a result, more than half of an average *Campylobacter* genome belongs to the core. However, other ways of computing gene families also use cutoffs in the same range, e.g. the 50-50 rule used by [17], corresponds roughly to a cutoff of 0.75 in our approach. Both [17], and [18] produced core size estimates for *Campylobacter* populations in a similar range. As seen in Figure 1, any choice of BLAST distance cutoff between 0.6 and 0.8 results in almost the same number of core gene families (less than 1% difference). With a smaller cutoff some of the gene families will have additional members from some genomes, but since we only include the ortholog from each genome in the downstream analysis, this will have no impact. The cutoff 0.8 maximizes the number of core gene families, which is our reason for choosing it. A too small cutoff will result in more gene families, but fewer core gene families since at least one genome will be lacking in some of the families obtained by cutoff 0.8. A too large cutoff will produce fewer core gene families because it produces too few gene families in the first place, by merging some of the gene families obtained by smaller cutoffs. The cutoff 0.8 obtains the balance between these two effects for this data set.

### Principal components

The principal component analysis revealed that 60% of the variation in normalized evolutionary distances can be captured in three linear combinations (see Figure 2). This figure also indicates a substantial incongruence in the evolutionary distances for the various core gene families. If all genes displayed the same evolutionary signal, we would have captured all variability in a single principal component, i.e. 100% explained variance after the first component in Figure 2. The fact that the explained variance grows fairly slow means that the 1180 rows of the data matrix *X* contain many different patterns. We tried to build phylogenetic trees based on each CGF separately, and computed consensus-trees that indeed verified this (see Additional file 1: Figure S1). By considering only the three-dimensional principal component space, we are focusing our analysis on the major variability in the data. Performing the analysis in this subspace means the results are based only on the dominating evolutionary patterns, and all the smaller differences will be downweighted. Our use of PCA here will have an effect similar to the use of bootstrapping on phylogenetic trees, in the sense that only the dominating patterns in the data come to the surface.

It is clear from Figure 3 that most CGFs are found in a dense region near the origin, where 5 of the 7 MLST genes are also found. Apart from these, genes are mainly scattered to the right (along PC 1) or upwards (along PC 2) in the upper panel, or downward (along PC 3) in the lower panels. The loadings of Figure 4 indicate that the major variation in this data is related to the separation of *C.coli* from *C. jejuni*. Core genes with a small value in the first component coordinate (left side of Figure 3, upper panel) show a different separation of species than those with a large coordinate value (right side of Figure 3, upper panel). The remaining variation we have included (component 2 and 3) is highly influenced by three distinct genomes, *C. jejuni* 414, *C. coli* 6461 and *C. coli* 6067, from which the distances to all other genomes fluctuate severely.

### Core gene clusters

The cluster analysis reveals some clusters of genes that are distinctly separated from the majority. The gap-statistic analysis clearly indicates that going from *K*=1 to *K*=2 gives a large increase, indicating that this is not a homogeneous set of genes, and at *K*=5 we get the first peak, indicating that a partition of the CGFs into 5 clusters is optimal (see Figure 5).

These five clusters were further compared. The blue and the cyan clusters are just two parts of the same central set of CGFs. Merging these into one big group, it contains 935 of the 1180 *Campylobacter* core genes. Six of the seven MLST markers are in this main group, and in Figure 6 we can see that the consensus-tree for these genes separates all *C. coli* from the *C. jejuni* strains, but with *C. jejuni* 414 as a ’*coli*-like’ strain of *jejuni*. The red cluster in Figure 3 is mainly separated from the rest along the first principal component, which makes it the most distinct cluster outside the main group. The loading plot in Figure 4 suggests that this principal component has to do with the separation of the two species, and the consensus-tree of the red cluster in Figure 6 confirms this. Here *C. coli* strains are not separated from the majority of the *C. jejuni*. Hence, the 120 core genes in the red cluster tell a consistently different story about how all these strains are related compared to the blue cluster. Also, note that the MLST marker *aspA* as well as the marker PorA are in this red group. The green cluster in Figure 3 is located at the same position along PC1 as the blue group, and is only separated along the second component. The green consensus-tree is also quite similar to the blue, but with the noticeable difference that for these 103 core genes *C. coli* 6067 is no longer found in the *C. coli*-branch. This is in essence the effect of the second principal component, as was also indicated in Figure 4 (distances to *coli* 6067 are different). Finally, the small brown cluster, which is only separated along the third component, has a consensus-tree that is a mixture of the red and the blue tree. The PC3-typical information, which is related to the strains *jejuni* 414 and *coli* 6461 is not strong enough to affect the consensus-tree in Figure 6.

Many tests for phylogenetic congruence are designed to compare neighboring sequences on the chromosome (sequence ’windows’) and breakpoints are identified that may correspond to recombination events. Our search for gene clusters is not using the positional information, but as shown in Table 2, the clusters we find are still highly enriched by neighboring genes. The fact that all groups show a clumping index *I* larger than 1.0 indicates that core genes are themselves not a random selection of genes in the reference genome (*C. jejuni* 11168 was arbitrarily chosen, see Methods). The three groups we identify outside the main group (colored red, green and brown in the figures) all have a very large clumping index. Thus, the genes within these clusters are very often found next to each other on the chromosome.

We also found that among those genes showing indication of being under selective pressure, 28 out of 30 are in the red or brown cluster (Table 2). These two clusters deviate from the other CGFs by their location along the PC1 direction which, as can be seen from Figures 4 and 6, represents the separation of species. A large score along PC1 means less separation between *jejuni* and *coli*, and this seems to coincide with selection pressure.

The computation of the population recombination rate *γ* is another descriptor of the the CGFs. CGFs with a large *γ*value are indications of loci with HGT contributing to increased genetic variation. From Figure 7 and Table 2 we see that again the red cluster separates from the blue main group by having on average an almost twice as large recombination rate. Also the green cluster tends to have slightly larger *γ* values, but this increase is just weakly significant (p=0.02).

In [11] indications of convergence between the two sympatric sister species *C. jejuni* and *C. coli* were found, based on analysis of a large number of MLST isolates. These results have later been countered in a re-analysis by [19], and in a pangenome study by [18] it was also concluded there is no evidence of convergence between these two species. Lefebure *et al.* found that a total of 80% of the core genes were free of any between-species recombination, and even if we have made no attempt of tracing the history of any recombination events, our results show that 89% of the core genes maintain a good separation of the two species (blue/cyan and green clusters). Also, our interpretation of the first, and most important, principle component as a species separation means our results support the conclusion in [18] with respect to convergence of the species.

## Conclusions

To be clonal is to have a single common ancestor uncluttered by horizontal gene transfer. In a clonal or weakly clonal situation the only factor that should determine the evolutionary distances between alleles is time. If this was the case for *Campylobacter*, there should be only one focal cloud in the score plot in Figures 3, with a completely stochastic variation around the center. Instead of this, we observe clusters along the principal component directions, and these groups seem to be far from random. Especially the red cluster, which is separated from the rest along the most important principal direction, is also characterized by many genes under selective pressure and with high recombination rates. This is the expected finding of a population with a mixture of evolutionary patterns, also known as a mercoclone.

The creation of clusters in the PCA can have multiple explanations for situations that may or may not involve HGT. The key is that there is an apparent change in the mutation rate that is uniform across some loci, creating a distinct cluster in the score plot. Deviations from a ’normal’ rate can be caused by a strong selection for diversity. The genes with the same selection forces should have similar evolutionary patterns and therefore be in clusters, each cluster reflective of the selective force. This seems to be the case for the red, and possibly the brown, cluster here. Clusters could also reflect transfer of alleles for different loci from similar sources at about the same time. However, this effect should be stronger in the short term, and expected to be diluted away over time if all the loci are equally subject to HGT.

A phylogenic analysis is aimed at telling the story of the ancestral derivation of modern clones. Different phylogenies tell different stories and when there are incongruent phylogenies for genes used in MLST analysis it is usually assumed that horizontal gene transfer has brought together genes with different ancestries. The principal component analysis that we have employed here clearly indicates that the set of core genes in *Campylobacter* cannot be seen as a single group of phylogenetic markers, but contains at least two, possibly five, distinct groups of genes carrying different signals on how *Campylobacter* strains have evolved.

## Methods

### Genome sequences used in this analysis

A total of 27 sequenced *Campylobacter* genomes from 22 *C. jejuni* and five *C. coli* isolates were included for analysis. Plasmid sequences were excluded. Nine of the genomes were completed and accessible at NCBI whereas 14 were available in draft form at the time of analysis (http://www.ncbi.nlm.nih.gov/genomes/lproks.cgi). In addition, four genomes were included that have not yet been publicly released. Since available annotations had been produced by various research groups using different protocols, all genes in all 27 genomes were re-defined using the software Prodigal v2.0 [20] for the sake of completeness and standardization. Although it is not suggested that this software is performing better than others, standardized gene finding overcomes the introduction of differences introduced by different gene finders. Moreover, since this analysis concentrates on conserved core genes only, re-annotation is not thought to cause inaccuracies.

### Identification of core gene families (CGF)

In order to compute gene families and identify conserved core genes, all predicted proteins in each genome were compared by BLASTP to all other proteins and a BLAST distance metric between every pair of sequences was computed. Let *S*(*a*;*b*) be the largest BLAST alignment bitscore for aligning sequence *a* against *b*, using *a* as the query. Then the BLAST distance is defined as

(1) $$B(a,b)=1-\frac{1}{2}\left(\frac{S(a;b)}{S(a;a)}+\frac{S(b;a)}{S(b;b)}\right)$$

This distance, which is a simple approximation to an evolutionary distance between two genes, ranges from 0 when perfect identity exists between *a* and *b*, to 1 in case no BLAST hit could be identified. Using these distances, gene sequences were grouped by a single linkage graph clustering algorithm, using the igraph package in the R computing environment (http://www.r-project.org). Every sequence was represented by a node in a graph, and nodes were connected if their pairwise BLAST distance is less than 1. All disconnected sub-graphs thus provided the first approximate sequence clusters. Next, in each of these clusters, genes were grouped by hierarchical clustering using complete linkage [21]. Finally, sequences were clustered from the resulting dendrogram by using a defined BLAST distance cutoff. The choice of cutoff determines the tightness of the gene families, and thereby also the number of core gene families (CGF).

Some genomes may contribute multiple gene members in a CGF and in such cases we only included the gene producing the smallest sum of distances to all other group members. This most likely corresponds to eliminating paralogs from the gene families, resulting in exactly 27 members (most likely orthologs) in each CGF. Using the protein sequences of these orthologs, a multiple alignment was computed for each CGF using the software M-Coffee [22]. This combines several multiple alignment tools, and builds a final alignment as a weighted consensus, making the result less dependent on the heuristics of any single algorithm. Next, for every alignment sequences were de-translated back to DNA using the TranslatorX software [23], and this DNA-alignment was pruned by the Gblocks software [24] to eliminate non-informative positions with too many gaps.

### Evolutionary distances

Based on the multiple alignments, an evolutionary distance table between matching CGFs was computed for all the genomes. Multiple substitutions were corrected for using the model of Tamura and Nei [25] with a gamma correction. Other evolutionary models were also tried, all of which produced essentially identical results in the final analyses.

For each CGF a 27×27 distance table was produced. Dividing the numbers in each distance table by its mean value, we get a set of normalized evolutionary distances. This normalization means we remove the absolute dissimilarity between genomes, and only consider relative differences. Two CGFs, one with large and one with small differences between the genomes, will be considered similar if the relative difference between the genomes is the same. For CGF *i* all distances in the lower triangle of the normalized evolutionary distance table were put into the row-vector _***x****i*_ in a fixed order. These row-vectors were assembled into a matrix

(2) $$X=\left[\begin{array}{c} x_{1,1}\ldots x_{1,351}\\ \;\;\;\vdots \;\ldots \;\;\vdots \\ x_{n,1}\ldots x_{n,351} \end{array}\right]$$

for *n* CGFs. With 27 genomes in our data set there are 27·26/2=351 unique distances for each CGF. Hence, the matrix ***X***is an *n*×351 matrix, where *n* is the number of CGFs used. Every CGF is a point in this 351-dimensional space, and those who are close to each other in this space describe the difference between the 27 genomes in a similar way.

### Principal components and partitioning

In order to reduce the dimensionality and remove unimportant variability in the evolutionary space we used a principal components analysis. This means we decompose the *n*×351 matrix ***X*** as

(3) X=ZL+E

where the ***Z*** is the *n*×*q* score matrix, ***L*** is the *q*×351 loading matrix and ***E*** is the remaining variation in ***X***. The main idea behind PCA is to choose a small value for *q*, e.g. *q*=2, which means the 351 coordinates for each row in ***X***is instead approximated by the *q* coordinates of the corresponding row in ***Z***, and all remaining dimensions are truncated under the assumption they contribute mainly with noise. A score plot will show each row of ***Z***as a point in a *q*-dimensional space. A loading plot will show each of the 351 columns of ***L***, one for each of the original columns of ***X***, in a similar way.

Central to the meroclone-hypothesis is the presence or absence of clusters of the core genes in the evolutionary space. To investigate this we used the k-means clustering method together with the gap-statistic [26,27]. The gap-statistic is a way of testing for the natural number of groups in a data set. Using k-means we partitioned the data into *K*=1,2,…,10 clusters, and for each value of *K* we computed the gap-statistic. The optimal number of clusters is the smallest *K* where we see a significant drop in the gap-statistic. In a weakly clonal polpulation we expect *K*=1 to come out as optimal, i.e. all genes belong to the same group.

### Gene features

From the core gene sequences we also derived some additional gene features. In case the PCA indicates certain groupings or patterns, it is always preferable to be interpret these in the light of other characteristics of the genes. Any type of grouping which is also meaningful from another viewpoint is less likely to be an artifact.

#### Physical position

Using the reference genome *jejuni* NCTC 11168 we ordered all predicted genes (also those not member of a CGF) from 1 to 1658 (there are 1658 predicted genes in *jejuni* NCTC 11168) beginning at the replication initiation. For any selection of a pool of genes of size *m* we counted the number of neighbors on the chromosome within this group. The positional distribution of a random selection of size *m* can be approximated by a Poisson process, and the physical distance between the genes as waiting times in this process. This follows an exponential distribution and the probability of neighborhood between two consecutive genes is *ρ*=1−exp(−*λ*) where *λ*=*m*/1658. For each grouping of genes of size *m* we computed the ’clumping’ index *I* as

(4) $$I=\frac{N}{\mathrm{m\rho}}$$

where *N* is the observed and *mρ*is the expected number of neighbors in the group of size *m*. If *I* is (much) larger than 1 it indicates the genes in the group are more often neighbors than expected by random chance.

#### Selective pressure

Based on the multiple alignments for each core gene family we computed the Tajima’s *D* statistic [28] which is an indicator of the selective pressure acting on a gene. Genes with Tajima’s *D* values significantly different from zero (*p*=0.05) were categorized as under selection. The remaining genes have selectively neutral evolution, i.e. genetic drift. For any group of genes we used the Fisher exact test to test for enrichment of genes under selective pressure within the group.

#### Recombination

From the multiple alignments we also computed the parameter *γ* as an estimate of population recombination rate [29] based on data for each CGF. A larger value of *γ*indicates a larger production of genetic variation at the corresponding locus.

## Competing interests

The authors declare that they have no competing interests.

## Authors’ contributions

RJM and TMW conceived of the study. LS carried out most data analysis and programming, RJM and KL collected and computed the gene features. EA, JO, SKa, SKn, MT and DWU provided unique data sets. LS, TMW and RJM drafted the manuscript, and all co-authors contributed to the discussion of the results. All authors read and approved the final manuscript.
